# Daytime and nighttime glycemic control with control-IQ technology vs. standard therapy in type 1 diabetes: a systematic review and meta-analysis with trial sequential analysis and GRADE assessment

**DOI:** 10.1186/s13098-025-01906-2

**Published:** 2025-08-12

**Authors:** Rahma Mogahed Rateb, Ammar Salah, Ahmed Kertam, Youssef Adel Youssef Ashmawi, Nourhan Hatem Mahmoud, Eslam Afifi, Ahmed Bayoumi, Salma Allam, Mohamed Saad Rakab

**Affiliations:** 1 Faculty of Medicine, Assiut University, Assiut, Egypt; 2 Faculty of Medicine, Al-Azhar Assiut University, Assiut, Egypt; 3 Faculty of Medicine, Ain-shams University, Ain-shams, Egypt; 4 Faculty of Medicine, October 6 University, Giza, Egypt; 5 Faculty of Medicine, Suez Canal University, Ismailia, Egypt; 6 Faculty of Medicine, Benha University, Benha, Egypt; 7 Faculty of Medicine, Galala University, Suez, Egypt; 8 Faculty of Medicine, Mansoura University, Mansoura, Egypt

**Keywords:** Control IQ, Artificial pancreas, Closed loop, Type 1 diabetes

## Abstract

**Background:**

Automated insulin delivery showed better results than standard insulin therapy, such as MDI. Therefore, we aimed to discuss the efficacy of Control IQ hybrid closed loop (HCL) during the daytime and nighttime for the type 1 diabetic population without restriction in age group or disease severity.

**Methods:**

We searched PubMed, Scopus, Cochrane Library, Web of Science, and related article citations. We analyzed the time in range (TIR), time below range (TBR), time above range (TAR), HBA1c, and serious adverse events (AEs) to ensure safety and efficacy. We used a random effects model when heterogeneity was present; otherwise, we used a fixed effects model. Data was presented as mean difference and 95% confidence intervals.

**Results:**

We selected seven randomized controlled trials (RCTs) out of 1339 articles for the analysis. After analysis of pooled studies data, Control IQ showed improvement in TIR 70–180 mg/dl 24 h data (MD 11.75%, CI 95% (9.54 to 13.97), *p* = 0.000001) with a similar effect observed in daytime and nighttime. Significant reduction in HbA1c (MD -0.38%, CI 95% (-0.55 to -0.22), *p* = 0.00001) and 24-hour coefficient variation (CV) measurement (MD -1.42%, CI 95% (-2.22 to -0.61), *p* = 0.0006) in the Control IQ group compared to the standard therapy group. However, daytime TBR < 70 mg/dl did not show significant improvement (MD -0.22%, CI 95% (-0.50 to 0.06), *p* = 0.12). Regarding safety, Control IQ reduction in DKA was insignificant (OR 1.48 (CI 95%: 0.23–9.55), *p* = 0.68).

**Conclusion:**

Control IQ showed better blood glucose control than standard insulin therapy, with some considerations regarding daytime glycemic control that need further assessment.

**Supplementary Information:**

The online version contains supplementary material available at 10.1186/s13098-025-01906-2.

## Introduction

Diabetes mellitus (DM) is one of the most prevalent chronic disorders affecting millions of people worldwide [1]. The treatment of DM is often complicated, with multiple approaches to treatment involving lifestyle modifications, many different oral hypoglycemic drugs, and various types of insulin regimens [2]. Therefore, maintaining compliance and efficacy in treating diabetes is a significant challenge. Ineffective treatment can result in poor blood glucose control, leading to many dangerous complications, such as hypoglycemia, coma, and organ damage [3].

Automated insulin delivery (AID) systems have been developed to achieve better glycemic control over prolonged periods, reduce the incidence of complications, and improve quality of life [4]. One example of an AID system is the hybrid-closed loop (HCL) system, which can adjust the basal insulin level automatically based on a computer-engineered algorithm; however, it has the limitation of requiring manual adjustment of bolus doses [4, 5].

Tandem t: slim X2 insulin pump with Control-IQ technology (Control-IQ) is an advanced hybrid-closed loop system (ACHL) that is considered an improvement over the usual HCL systems as it can adjust both the basal and bolus insulin doses automatically [6]. It consists of an insulin pump based on Control-IQ technology, a continuous glucose monitoring (CGM) device that provides real-time glucose readings, and an algorithm capable of adjusting the insulin dose automatically based on the glucose readings [7]. It also offers flexibility in glycemic control, as it can adjust multiple desired glucose levels for the device to sustain, unlike other HCL devices that use a fixed glucose target level [6, 7]. A previous meta-analysis based on three randomized controlled clinical trials (RCTs) reported that Control-IQ increased the percentage of patients in Time in Range 70–180 mg/dL (TIR) compared to control and also proved the superiority of Control-IQ in other secondary outcomes [7]. We reviewed the literature and found that other clinical trials have not been reported in the previous meta-analysis, which could affect the ability to generalize the results, especially in patients with a high risk of hypoglycemia [6–10].

Therefore, we conducted this systematic review and meta-analysis to explore the efficacy and safety of Control-IQ technology compared with usual care (continuous insulin infusion, multiple-dose injection, or sensor-augmented pump) using a larger sample size obtained from previous RCTs.

## Methods

### Data sources

We performed the meta-analysis agreeing to PRISMA guidelines and the Cochrane Handbook of Systematic Reviews and Meta-Analysis [11, 12]. This review was registered in PROSPERO (CRD42024576521).

### Search strategy

We searched PubMed, Scopus, Web of Science, and the Cochrane Central Register of Controlled Trials until July 2024. The search terms used were “Tandem Control IQ” and “Advanced Hybrid Closed Loop”. Moreover, we manually searched the reference lists of included studies and the relevant reviews. The detailed search strategy was presented in (Supplementary Table 1).

### Eligibility criteria

We included randomized controlled trails (RCTs) reporting the following criteria: (1) population; type 1 diabetes patients, (2) intervention; Control IQ technology closed loop system, (3) comparator; standard insulin therapy as multiple daily insulin injections (MDI), continuous subcutaneous insulin infusion (CSII), and sensor-augmented pumps (SAP) with or without low glucose suspension, (4) Outcomes: time in range (TIR) 70–180 mg/dl.

We excluded animal studies, duplicate datasets, books and not English language articles.

### Study selection

Records identified from our search were imported into Rayyan, and duplicate references were removed [13]. We initially screened the titles and abstracts and then the full text by four authors independently (NH, YA, RM, and EA). The investigator (MS) resolved conflicts.

### Data extraction

Four reviewers (NH, AS, AB, and YA) independently extracted data from each report according to the pre-decided data extraction sheet. We extracted [1] characteristics of the included studies: study design, total number of participants, country, population, comparator, follow up, timing, and setting; [2] baseline data of the participants: age, BMI, gender, diabetes duration, past history of severe hypoglycemia events, past history of diabetic ketoacidosis events, Hba1c level, and total daily insulin dose; [3] outcomes. We extracted data for 24-hour, daytime and nighttime endpoints whenever possible; we presented the extracted endpoints in (Supplementary Table 2). The final sheet was reviewed by a third investigator (RM).

### Outcome measurements

Our study primary outcomes were the time in near euglycemic ranges (TIR), which are defined as the percentage of the intervention time the blood glucose was in the range of (70–180 mg/dl) and (70–140 mg/dl). The secondary outcomes were the time below range (TBR) at different levels (< 70 mg/dl and < 54 mg/dl), the time above range (TAR) in different levels (> 180 mg/dl and > 300 mg/dl), the coefficient of variation and mean blood glucose levels, glycated hemoglobin, and number of patients with HbA1c value less than 7% at the end of the trial; for studies with a T1D management duration of 2 months, high blood glucose index (HBGI), low blood glucose index (LBGI), severe hypoglycemia events as defined by each study, and diabetic ketoacidosis.

### Quality assessment

Four reviewers (NH, AS, AB and YA) evaluated the quality of RCTs independently using the Cochrane risk of bias tool version 2 (ROB 2) [14]. This tool evaluates the following five domains: assessment of the randomization process, achievement of intervention control per the study plan, absence of outcome data, outcome measurement, result reporting, and overall risk of bias. Studies were classified as low risk when all domains were low risk and high risk when at least one domain was high risk or three domains were some concerns. The GRADE (Grading of Recommendations Assessment, Development and Evaluation) framework was employed to assess the quality and strength of the evidence [15]. All disagreements were reconciled through discussion with a senior author (MS).

### Statistical analysis

We used RevMan 5.4.1 software to do the meta-analysis [16]. We reported continuous outcomes as mean difference (MD) and dichotomous outcomes as odds ratio (RR); for all outcomes, a confidence interval of 95% was calculated. We used the last time point data on the analysis. We converted study data reported as median (IQR) to mean (SD) when the SD was not directly reported [17]. We assessed heterogeneity by the Chi-square (I²) test, and a *p*-value less than 0.1 was considered significant. According to Cochrane guidelines an I^2^ value of 0–40% indicating low heterogeneity, 30–60% indicating moderate heterogeneity, 50–90% possibly representing substantial heterogeneity, and 75–100% signifying considerable heterogeneity. We used a random effects model when heterogeneity was found; otherwise, we used a fixed effects model. When the *P*-value is less than 0.05 the results were statistically significant. We conducted a sensitivity analysis to fix the heterogeneity and evaluate the impact of these studies on the effect size. Sensitivity analysis was applied to studies with short follow up duration and studies reporting the population with severe hypoglycemia risk to assess robustness of the synthesized results. We could not do a publication bias analysis using the forest plot as the number of studies included was less than ten [18]. We conducted the trial sequential analysis to assess reliability of our results. The level of confidence for the intervention is sufficient and conclusive when the cumulative z-line on the curve crosses the sequence monitoring border, meaning that no additional research is needed [19]. We conducted the trial sequential analysis using TSA software version 0.9.5.10 Beta. We used a conservative Required information size (RIS) if there was a technical limitation of graph report formation by the Automated TSA software built in function which incorporate anticipated effect size, Type 1 and II levels and diversity from the included studies.

## Results

### Literature search

**Fig. 1:**
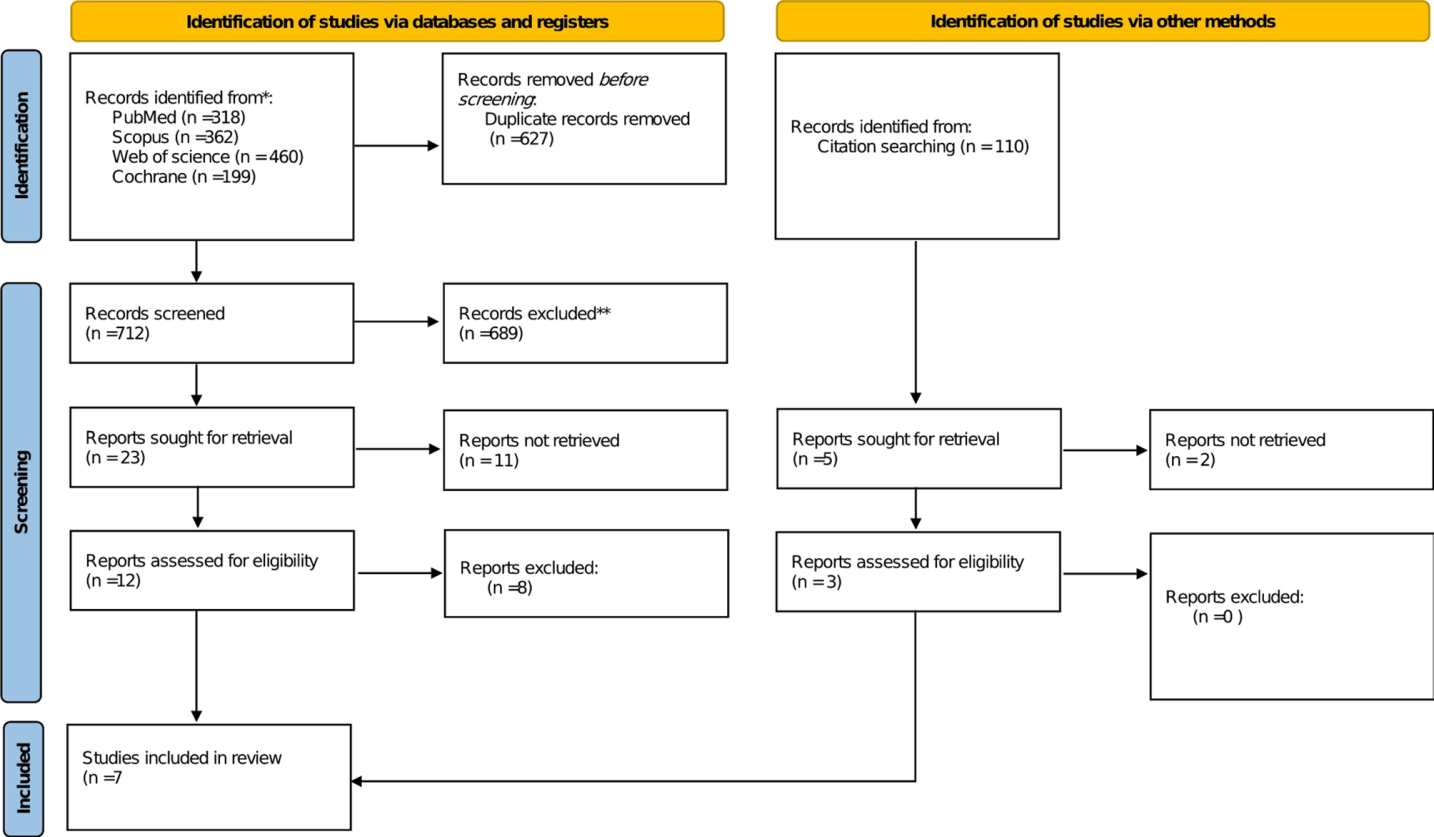
PRISMA flow diagram

A literature search of the four included databases, revealed 1339 citations, we incintified 627 as duplicates. After the removal of duplicates, 712 articles entered title and abstract screening. Twenty-three were valid for full-text screening, and seven clinical trials were involved in this study, as illustrated in the PRISMA flow diagram (Fig. 1).

### Baseline characteristics of included studies

Across seven trials, 624 patients were included, with 397 patients in the Control IQ group and 297 patients in the control group [6, 8, 9, 20–23]. All studies were conducted in US with SAP mostly used in control group. The comparison between the two groups did not reveal any significant differences. (Tables 1 and 2).

**Fig. 2:**
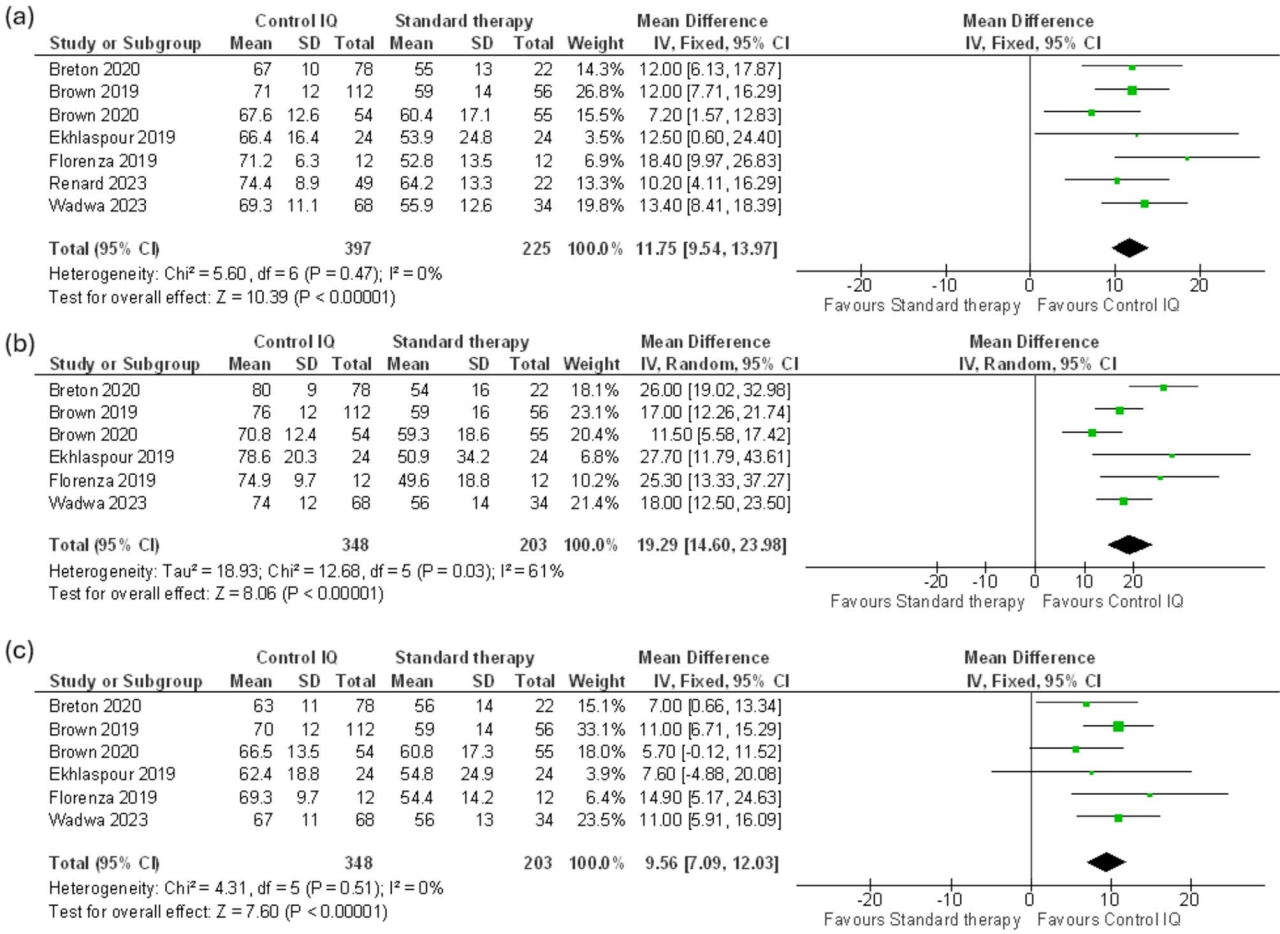
(**a**) Time in range (TIR) 70-180 mg/dl, (**b**) Nighttime TIR 70-180 mg/dl, (**c**) Daytime TIR 70-180 mg/dl

**Table 1:** Study characteristics. RCT: randomized controlled trial, SAP: sensor-augmented pump, PLGS: predictive low glucose suspension

Study ID	Study design	Total number	Country	Population	Comparator	Follow up	Timing	Setting
Renard 2023	Parallel-arm RCT	72	US	Adults with T1D and a high risk for severe hypoglycemia	SAP/PLGS	12 weeks	24 h	Outpatient
Wadwa 2023	Parallel-arm RCT	102	US	Children with T1D	MDI/SAP/CSII/PLGS	13 weeks	24 h	Outpatient
Breton 2020	Parallel-arm RCT	101	US	Children with T1D	SAP/PLGS	16 weeks	24 h	Outpatient
Brown 2019	Parallel-arm RCT	168	US	Children and adults	SAP	26 weeks	24 h	Outpatient
Ekhlaspour 2019	Parallel-arm RCT	54	US	Children and adolescents	SAP	48 h	24 h	Inpatient
Florenza 2019	Parallel-arm RCT	24	US	School-aged children (6–12 years)	SAP	three days	24 h	Outpatient
Brown 2020	Parallel-arm RCT	109	US	Children and adults	PLGS	13 weeks	24 h	Outpatient

**Table 2:** Baseline data. BMI: body mass index, DKA: diabetic ketoacidosis, continuous data presented as mean (SD) and dichotomous data as number (percent)

Study	Age (years) (Control IQ / Standard therapy)	BMI (kg/m²) (Control IQ / Standard therapy)	Male (Control IQ / Standard therapy)	Diabetes Duration (years) (Control IQ / Standard therapy)	Past Severe Hypoglycemia Events (Control IQ / Standard therapy)	Past DKA Events (Control IQ / Standard therapy)	HbA1c (mmol/mol) (Control IQ / Standard therapy)	Total Daily Insulin Dose U/kg (Control IQ / Standard therapy)
Renard 2023	47.2 (12.5) / 47.1 (13.3)	26.0 (4.7) / 25.8 (3.1)	15 (30) / 12 (52)	28.2 (13.2) / 27.2 (12.2)	13 (56) / 23 (47)	N/A / N/A	7.3 (0.6) / 7.1 (1.6)	0.6 (0.2) / 0.6 (0.2)
Wadwa 2023	3.84 (1.23) / 4.06 (1.25)	NA / NA	35 (51) / 15 (44)	1.2 (0.86) / 1.47 (0.93)	4 (6) / 2 (6)	11 (16) / 3 (9)	7.5 (1.2) / 7.7 (0.9)	NA / NA
Breton 2020	11.3 (2.0) / 10.8 (2.4)	NA / NA	40 (51) / 11 (48)	5.0 (2.8) / 6.0 (2.8)	0 / 0	0 / 0	7.6 (1.0) / 7.9 (0.9)	0.89 (0.24) / 0.94 (0.24)
Brown 2019	33 (16) / 33 (17)	25.7 (4.5) / 25 (4.6)	58 (52) / 26 (46)	17.7 (15.01) / 15 (12.01)	6 (5) / 1 (2)	4 (4) / 1 (2)	7.4 (1.0) / 7.4 (0.8)	NA / NA
Ekhlaspour 2019	12.5 (3.1) / 12 (3.2)	20.4 (2.8) / 21.7 (5.7)	13 (54) / 11 (46)	5.7 (2.9) / 5 (2.0)	0 / 0	0 / 0	7.7 (0.9) / 7.8 (1.3)	0.8 (0.2) / 0.8 (0.2)
Florenza 2019	10 (2.1) / 9.2 (1.5)	19.2 (2.7) / 17.8 (3.5)	6 (50) / 6 (50)	4.7 (2.3) / 4.4 (1.4)	0 / 0	0 / 0	7.35 (0.74) / 7.36 (0.65)	0.76 (0.21) / 0.71 (0.18)
Brown 2020	32 (14) / 34 (17)	26.3 (5.3) / 25.6 (4.5)	26 (48) / 30 (55)	18.3 (16) / 18 (18.3)	NA / NA	NA / NA	7.9 (0.9) / 7.6 (1.0)	0.65 (0.28) / 0.69 (0.36)

### Risk of bias of included studies and GRADE assessment

The ROB-2 tool was employed to assess risk of bias. Four trials showed low risk of bias, while two studies were evaluated as high risk of bias and one showed some concerns due to deviations from intended interventions (Supplementary Fig. 1). The Grade assessment indicated that certainty of evidence ranged from moderate to very low (supplementary Tables 3&4).

### Primary outcomes

#### Time in range TIR 70–180 mg/dl

Our meta-analysis revealed that the Control IQ showed a significant increase in the percentage of time spent in the 70–180 mg/dl range in 24-hour time (MD 11.75 mg/dl, CI 95% (9.54 to 13.97), *p* = 0.000001) with no heterogeneity (I^2^ = 0%, *P* = 0.47) (Fig. 2a). Across six trials that reported nighttime TIR 70–180 mg/dl data of 548 patients, significant results favored the Control IQ (MD 19.29%, CI 95% (14.6 to 23.98), *p* = 0.00001) with substantial heterogeneity (I² = 61%, *P* = 0.03). (Fig. 2b). We best-fixed heterogeneity by excluding Brown 2020 (MD 20.81%, CI 95% (16.55 to 25.08), *p* < 0.0001), (I^2^ = 39% *p* = 0.16) (Supplementary Fig. 2). Across the same six trials, daytime TIR 70–180 mg/dl data indicated significant results (MD 9.56%, CI 95% (7.09 to 12.03), *p* = 0.00001) with no heterogeneity (I² = 0%, *P* = 0.51) (Fig. 2c).

#### Time in range TIR 70–140 mg/dl

**Fig. 3:**
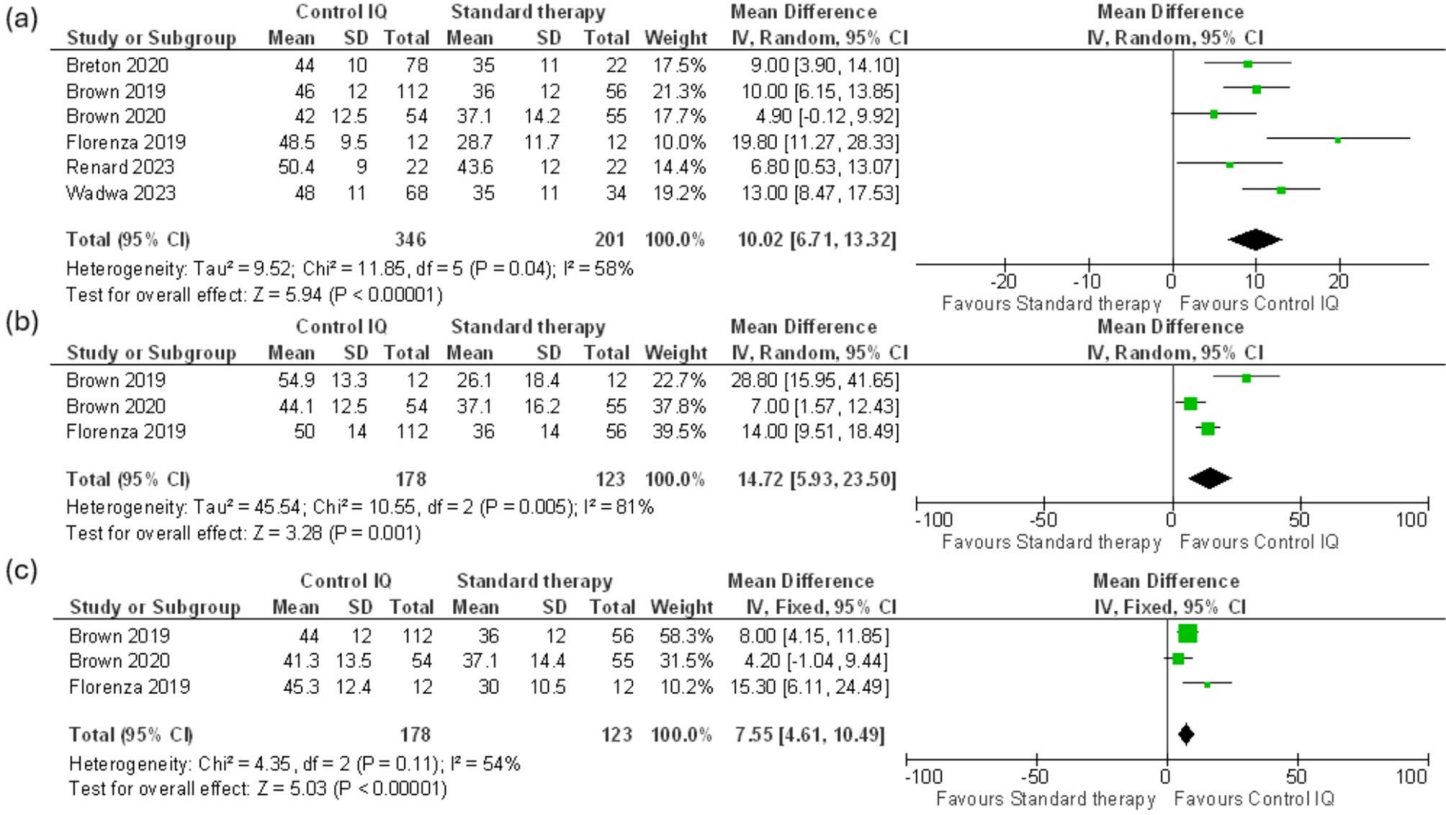
(**a**) Time in range (TIR) 70-140 mg/dl, (**b**) Nighttime TIR 70-140 mg/dl, (**c**) Daytime TIR 70-140 mg/dl

Regarding Time in the 70-140 mg/dl range, the outcome was reported in six trials with 547 patients. The analysis of 24-hour data highlighted a considerable difference between Control IQ and Standard therapy (MD 10.02%, CI 95% (6.71 to 13.32), *p* = 0.00001) with substantial heterogeneity (I² = 58%, *P* = 0.04) (Fig. 3a). We resolved the heterogeneity by discarding Florenza 2019 (MD 9.04%, CI 95% (6.33 to 11.75), *P* < 0.00001). (I^2^ = 36% *P* = 0.18) (Supplementary Fig. 3). Across three clinical trials with 301 patients, nighttime 70-140 mg/dl data of Control IQ showed significant improvement (MD 14.72%, CI 95% (5.93 to 23.5), *P* = 0.001) with considerable heterogeneity (I^2^ = 81%, *P* = 0.005) (Fig. 3b). Across the same trials, daytime data of 70-140 mg/dl provided a significant improvement in patients who used Control IQ (MD 7.55%, CI 95% (4.61 to 10.49), *P* < 0.00001) with moderate heterogeneity (I² = 54%, *P* = 0.11) (Fig. 3c).

### Secondary outcomes

#### Time below range tbr < 70 mg/dl

**Fig. 4:**
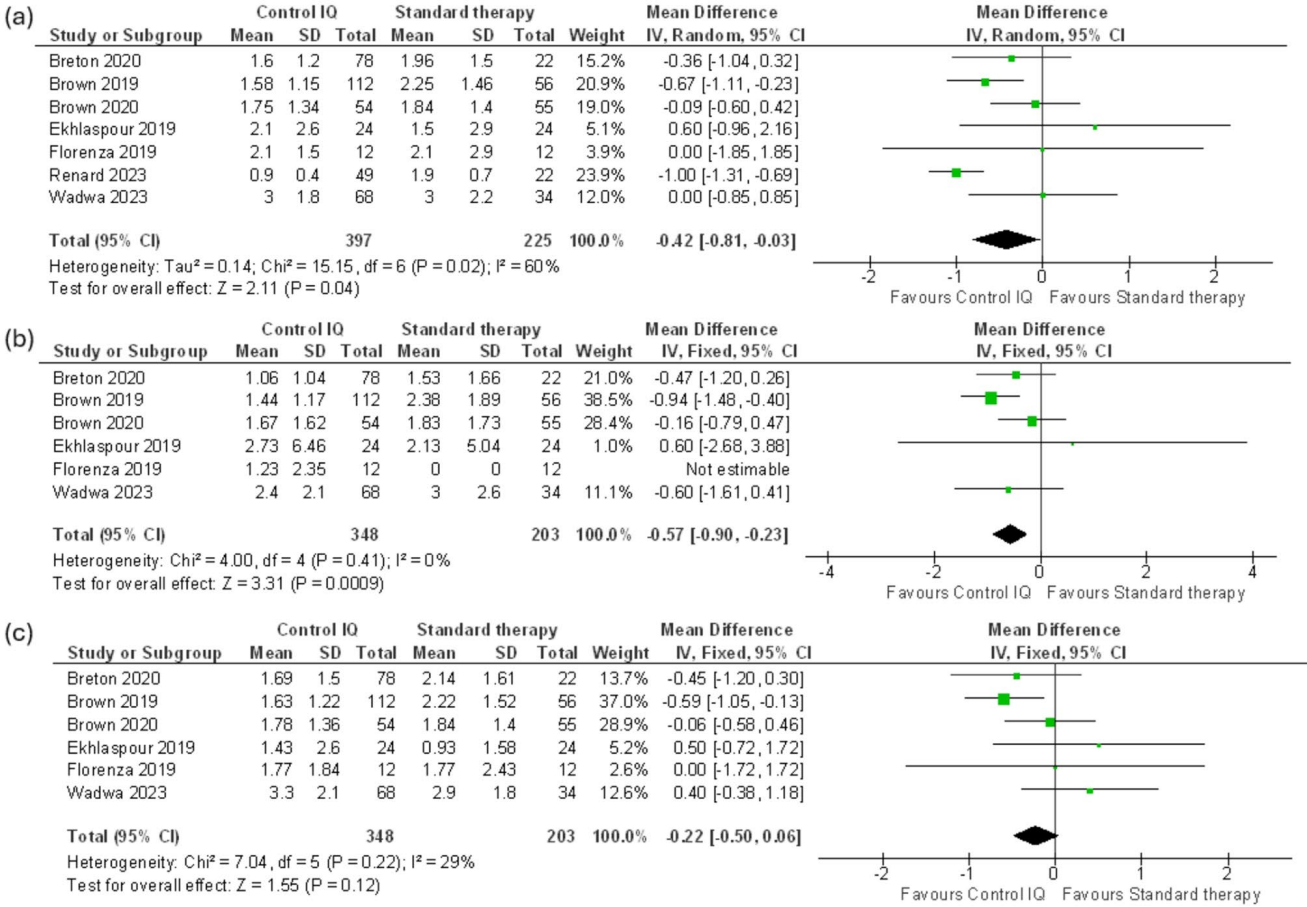
(**a**) Time below range (TBR) 70 mg/dl, (**b**) Nighttime TBR 70 mg/dl, (**c**) Daytime TBR 70 mg/dl

Our meta revealed that Control IQ successfully reduced time below range < 70 mg/dl (MD − 0.42%, CI 95% (-0.81 to -0.03), *p* = 0.04) with substantial heterogeneity (I^2^ = 60%, *P* = 0.02). (Fig. 4a). Heterogeneity was resolved by clearing Renard 2023 from the analysis (MD -0.32%, CI 95% (-0.61 to -0.04), *p* = 0.03). (I^2^ = 4%, *P* = 0.39) (Supplementary Fig. 4). The pooled analysis of the nighttime TBR < 70 mg/dl CGM measurements provided significant results (MD -0.57%, CI 95% (-0.90 to -0.23), *p* = 0.0009), the analysis revealed no heterogeneity (I^2^ = 0%, *P* = 0.41) (Fig. 4b). Across the same six trials, analysis of the daytime TBR < 70 mg/dl data analysis showed no significant improvement in the Control IQ. MD -0.22%, CI 95% (-0.50 to 0.06), *p* = 0.12) with low heterogeneity between the studies (I^2^ = 29%, *P* = 0.22) (Fig. 4c).

#### Time below range tbr < 54 mg/dl

**Fig. 5:**
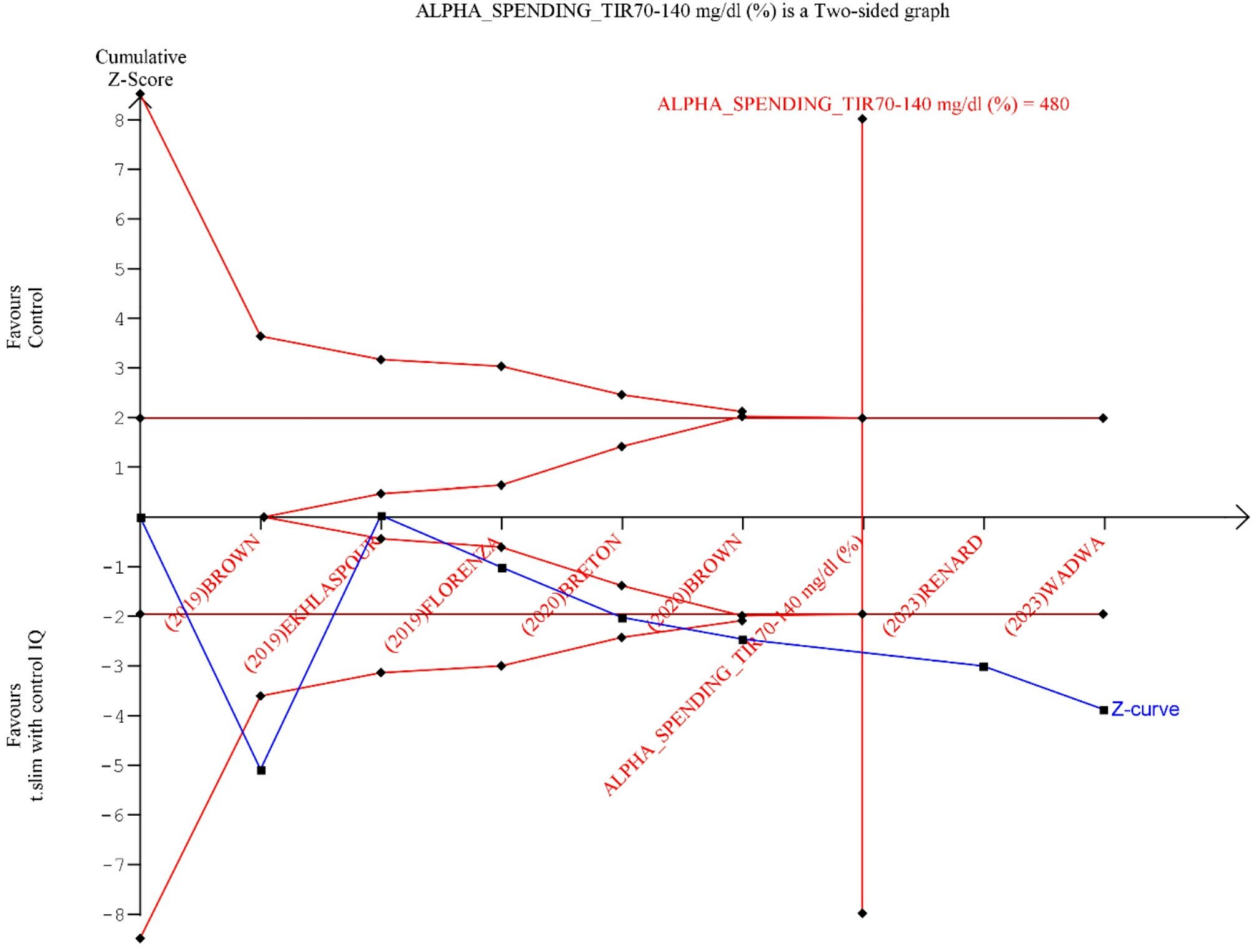
TIR 70–140 mg/dl trial sequential analysis, the required information size (RIS) to detect or reject the mean difference of 8.52 (C.I: 4.23 to 12.82) found in the Der Simonian-Laird (DL) model has been 311 participants using the Diversity found of 78%, with a double sided α of 0.05 and a β of 0.20 (power of 80.0%). We used a more conservative RIS of 480 to avoid a software limitation of graph report formation. The cumulative Z- curve (blue full line) passed both Superiority boundary and RIS sample line which suggest that the cumulative evidence is sufficient to conclude that Control IQ is superior to control group with the existing evidence

The analysis revealed no significant difference between the control group and the Control IQ group in the time below range TBR < 54 mg/dl (MD -0.07%, CI 95% (-0.19 to 0.06), *p* = 0.29) with a substantial heterogeneity (I² = 62%, *P* = 0.02) (Supplementary Fig. 5a). Heterogeneity is best resolved by filtering out Renard 2023 (MD -0.04%, CI 95% (-0.1 to 0.03), *p* = 0.27) (I^2^ = 0% *P* = 0.63) (Supplementary Fig. 6). Across four trials with 349 patients, nighttime TBR < 54 mg/dl CGM data showed an insignificant reduction in Control IQ (MD -0.02%, CI 95% (-0.21 to 0.16), *P* = 0.82) with substantial heterogeneity (I^2^ = 63%, *P* = 0.1) (Fig. 5b). Four trials reported the daytime TBR < 54 mg/dl data and our analysis found no significant improvement when using Control IQ compared to using standard therapy (MD -0.05%, CI 95% (-0.12 to 0.03), *P* = 0.2), with homogeneous results (I^2^ = 0%, *p* = 0.94) (Fig. 5c).

#### Time above range tar > 180 mg/dl

Our meta-analysis indicated that Control IQ favored Time above range TAR > 180 mg/dl outcome (MD -10.79%, CI 95% (-13.10 to -8.49), *P* < 0.00001) with low heterogeneity studies (I^2^ = 21%, *P* = 0.27) (Supplementary Fig. 7a). Nighttime TAR > 180 mg/dl data were reported by five trials with 449 included patients, and the results favored the Control IQ (MD − 19.35%, CI 95% (-25.62 to -13.08), *p* = 0.00001) with substantial heterogeneity (I^2^ = 67%, *P* = 0.02). (Supplementary Fig. 7b). We minimized the heterogeneity by omitting Brown 2020 (MD -21.79%, CI 95% (-27.79 to -15.59), *P* < 0.0001). (I^2^ = 49%, *P* = 0.12) (Supplementary Fig. 8). Daytime TAR > 180 mg/dl data analysis was significant too (MD − 7.98%, CI 95% (-10.97 to -5.00), *p* = 0.00001) with no heterogeneity (I^2^ = 0%, *P* = 0.59) (Supplementary Fig. 7c).

#### Time above range tar > 300 mg/dl

Our meta-analysis signified significant results (MD -2.43%, CI 95% (-3.29 to -1.57), *P* = 0.00001) with no heterogeneity (I^2^ = 0%, *P* = 0.94) in TAR > 300 mg/dl (Supplementary Fig. 9a). Across four trials, nighttime TAR > 300 mg/dl data showed significant results favoring Control IQ (MD -3.01%, CI 95% (-4.18 to -1.83), *P* = 0.00001) with no heterogeneity (I^2^ = 0%, *P* = 0.66) (Supplementary Fig. 9b). Regarding the daytime TAR > 300 mg/dl data, statistically significant results were observed (MD -2.03%, CI 95% (-3.25 to -0.82), *P* = 0.001) with no heterogeneity (I^2^ = 0%, *P* = 0.95) (Supplementary Fig. 9c).

#### Coefficient variation (CV)

Regarding CV, the results presented a significant difference between Control IQ and standard therapy (MD -1.42, CI 95% (-2.22 to -0.61), *p* = 0.0006) with moderate heterogeneity (I2 = 39%, *P* = 0.13) (Supplementary Fig. 10a). Across six trials, nighttime CV data analysis didn’t favor Control IQ (MD -1.57, CI 95% (-3.48 to 0.34), *p* = 0.11) with substantial heterogeneity (I2 = 67%, *P* = 0.01) (Supplementary Fig. 10b). We reduced heterogeneity by discarding Florenza 2019 (MD − 2.48, CI 95% (-3.76 to -1.2), *P* = 0.0002) (I2 = 31%, *P* = 0.21) (Supplementary Fig. 11). Daytime CV analysis results revealed significant results (MD -1.35, CI 95% (-2.21 to -0.49), *P* = 0.002) with no heterogeneity (I2 = 0%, *P* = 0.69) (Supplementary Fig. 10c).

#### Mean glucose

Across the seven trials with 624 patients, a meta-analysis of mean glucose results significantly favored the Control IQ (MD -14,18 mg/dl, CI 95% (-18.06 to -10.29), *P* < 0.0001) with low heterogeneity (I2 = 35%, *P* = 0.16) (Supplementary Fig. 10d).

#### LBGI

Five trials with 548 patients reported LBGI. The meta-analysis showed insignificant results (MD -0.15, CI 95% (-0.32, -0.02), *P* = 0.09) with considerable heterogeneity (I2 = 81%, *P* = 0.0003) (Supplementary Fig. 10e). We minimized the heterogeneity by dropping Renard 2023 (MD -0.08, CI 95% (-0.14 to -0.01), *p* = 0.03) (I2 = 9% *P* = 0.35) (Supplementary Fig. 12).

#### HBGI

Regarding the high blood glucose index, the outcome was reported in five trials, with 548 patients included. The analysis showed significant results (MD -2.25, CI 95% (-3.38 to -1.12), *p* = 0.00001) with moderate heterogeneity (I^2^ = 59%, *P* = 0.05) (Supplementary Fig. 10f). We reduced the heterogeneity to zero by excluding Brown 2020 (MD -2.8, CI 95% ( -3.64 to -1.95), *p* < 0.0001) (I^2^ = 0 *p* = 0.66) (Supplementary Fig. 13).

#### Hba1c

Meta-analysis showed a reduction in HbA1c in patients who used Control IQ (MD -0.38%, CI 95% (-0.55 to -0.22), *p* = 0.00001) with no heterogeneity (I² = 0%, *P* = 0.47) (Supplementary Fig. 14a). A meta-analysis of four trials showed a significant result for the number of patients with HbA1c percent less than 7% (OR 1.48, CI 95% (1.19 to 2.85), *p* = 0.006) with low heterogeneity (I² = 20%, *P* = 0.29) (Supplementary Fig. 14b).

#### Adverse events

There is no statistically significant difference in terms of side effects in both groups, (OR 1.48, CI 95% (0.23 to 9.55), *p* = 0.68) with no heterogeneity (I² = 0%, *P* = 1) and (OR 1.46, CI 95% (0.22 to 9.53), *p* = 0.69) with no heterogeneity (I² = 0%, *P* = 0.67) for DKA and severe hypoglycemic vents respectively (Supplementary Fig. 15a-b).

### Sensitivity analysis

We performed a sensitivity analysis for the TIR 70–180 mg/dl outcome. First, we excluded Florenza 2019 and Ekhlaspour 2019. These two studies have short follow-up durations; no differences were demonstrated (Supplementary Fig. 16). We also excluded Renard 2023 from the analysis. Renard 2023’s population was adults with T1D at a high risk of hypoglycemia, and the results showed significant results (Supplementary Fig. 17).

### Trial sequential analysis

To confirm our results, we conducted TSA for outcomes (TIR 70–140 mg/dl, TBR < 70 mg/dl, and mean glucose). If the graph showed that the cumulative Z curve crossed the conventional significance boundary the results would be conclusive, crossing the trial sequential monitoring boundary indicated sufficient evidence. TIR 70–140 mg/dl effect size was sufficient and conclusive as it crossed both TSA conventional and monitoring boundaries (Fig. 5). TBR 70 mg/dl and mean glucose results were shown in (supplementary Fig. 18–19).

## Discussion

Recent evidence shows that automated insulin delivery significantly improves glycemic control in 24 h. It provides a better lifestyle and less psychological burden [24].

The hybrid closed loop that used Control IQ technology was assessed in a prior meta-analysis by Beck et al. 2023. using three high-quality randomized controlled trials. We found subgroup analysis based on the study device reported by Godoi et al. 2023 and Jiao et al. 2022; these analyses showed limitations that led to the inclusion of studies not reporting Control-IQ exclusively [7, 25, 26]. However, all showed results favoring the Control IQ during the 24-hour time with some insignificant effects in reducing severe hypoglycemia. Our meta-analysis included seven trials with 624 patients using only insulin to compare Control-IQ technology with standard insulin therapy. Here, we reported its efficacy in daytime and nighttime in addition to 24-hour time.

The Control IQ improved TIR throughout the 24 h and had the best results in overnight measurements (MD = 18.17), which may be due to hormonal fluctuations during dawn phenomena and external factors like exercise and meals [27]. Control IQ overcomes fluctuations in blood glucose levels with the MPC algorithm, which delivers basal insulin boluses every five minutes and correction boluses [28]. These findings are consistent with those reported in prior meta-analyses [7].

The Control IQ improved TBR < 70 mg/dl in 24-hour time and nighttime measurements (*P* < 0.05) but showed insignificant improvement in the daytime measurements; this may be due to high exercise and daily work [29]. These results also can be attributed to the variability of insulin sensitivity throughout the day because of psychological changes [30]. We recommend using a higher blood glucose target in the daytime to minimize the risk of hypoglycemia [31]. The reduction in 24-hour, nighttime and daytime TBR < 54 mg/dl (level 2 hypoglycemia) was insignificant between the Control IQ and standard insulin therapy, these results is not comparable with the previous finding from the previous study [7].

Control IQ significantly decreased time spent in hyperglycemia TAR > 180 mg/dl in 24-hour, daytime and nighttime, with the highest reduction in nighttime (MD=-17.61); this may be due to fewer meal-related excursions, stressors that allow Control IQ algorithms to work best [32]. These results align with the outcomes observed in earlier meta-analyses [7].

Control IQ significantly decreased glycemic variability, reported as as CV in 24-hour time, and daytime, These findings are in agreement with those documented in the previous meta-analytic study [7]. The insignificant decrease in LBGI (*p* = 0.09) indicates insufficient benefit of Control IQ in reducing hypoglycemia episodes, especially overnight [33].

This meta-analysis showed a reduction in HbA1c due to decreased glycemic variability and increased TIR [32].

There was no significant reduction in total daily insulin intake; this can be attributed to the manual calculation of the meal bolus and user glycemic control variability with the primary goal of maintaining the patient in an euglycemic state [34].

Heterogeneity in outcomes reporting hypoglycemia was fixed by omitting Renard 2023; this was primarily due to the inclusion of a population with a high risk for hypoglycemia. The variety in TITR and overnight CV was fixed by omitting Florenza 2019 due to participants’ characteristics (primary school age children) or short study duration (3 days) or due to performance bias (patients in the SAP group were allowed to modify settings). Variability in HBGI, overnight TIR, and overnight TAR180 were fixed by omitting Brown 2020; this was primarily due to some patients transitioning from CLC to PLGM or due to performance bias (suspension of Control IQ for about four weeks). Additionally, discrepancies in continuous glucose monitoring (CGM) data collection and analysis, including the duration of CGM usage during the run-in phase. External factors such as training for the AID system and follow-up care may also differ across studies, influencing participant adherence and response to the intervention. These factors suggest that the trial that caused heterogeneity may have had unique methodological or participant-related characteristics, resulting in outliers in the results of some outcomes compared to others.

A limitation of this study is that different insulin-delivering modalities (MDI-CSII-SAP-PLGM) with different efficacy were pooled together in the standard insulin therapy group [35]. The definitions of nighttime and daytime varied among the trials, which may affect the results (Florenza 2019 et al. ranged from 23 to 7 h, which allowed one hour of dawn phenomena added to nighttime results) [36]. Nearly all the trials were conducted in the US, which limits the generalizability of the results. The trials excluded patients with a history of chronic renal disease or currently on hemodialysis, not adequately treated hypothyroidism, and bleeding disorder, which limits the clinical application of the results. All the included studies evaluate type 1 diabetes, so it cannot be applied to patients with type 2 diabetes. We converted data presented by median and interquartile ranges to median and standard deviation during data extraction. This data conversion allowed for a larger sample size but may have affected the data accuracy.

We recommend designing more clinical trials to explore the efficacy of Control IQ in reducing postprandial glycemic excursions and the risk of hypoglycemia in moderate to severe exercise. We encourage more trials that evaluate the efficacy of evening-night closed-loop control, which showed near glycemic benefit of the 24/7 CLC with uncertain superior daytime results [26].

## Conclusion

In conclusion, Control IQ insulin delivery is effective in maintaining the patient in a near-euglycemic state all day and preventing severe adverse events. However, there are some limitations in preventing a hypoglycemic state in the daytime, which needs more focus.

## Supplementary Information

Below is the link to the electronic supplementary material.

Supplementary Material 1

Supplementary Material 2

## Data Availability

No datasets were generated or analysed during the current study.
