# Supplementary material for: Daytime and nighttime glycemic control with control-IQ technology vs. standard therapy in type 1 diabetes: a systematic review and meta-analysis with trial sequential analysis and GRADE assessment
Source: Diabetol Metab Syndr. 2025 Aug 12;17:325. doi: 10.1186/s13098-025-01906-2 (PMC12341251; doi:10.1186/s13098-025-01906-2)
Supplement: Supplementary file 1 — Supplementary Material 1 [file 13098_2025_1906_MOESM1_ESM.docx]

**Daytime and Nighttime Glycemic Control with Control-IQ Technology vs. Standard Therapy in Type 1 Diabetes: A Systematic Review and Meta-Analysis with Trial Sequential Analysis and GRADE Assessment**

Rahma Mogahed Rateb^a^, Ammar salah^b^, Ahmed Kertam^c^, Youssef Adel Youssef Ashmawi^d^, Nourhan Hatem Mahmoud^e^, Eslam Afifi^f^, Ahmed Bayoumi^d^, Salma Allam^g^, Mohamed Saad Rakab^h^.

*^a^Faculty of Medicine, Assiut University, Assiut, Egypt ; ^b^Faculty of Medicine, Al-Azhar Assiut University, Assiut, Egypt****; ^c^****Faculty of medicine, Ain-shams University, Ain-shams, Egypt; ^d^Faculty of Medicine, October 6 University, Giza, Egypt; ^e^Faculty of medicine Suez Canal University, Ismailia, Egypt; ^f^Faculty of Medicine, Benha University, Benha, Egypt; ^g^Faculty of Medicine, Galala University, Suez, Egypt; ^h^Faculty of Medicine, Mansoura University, Mansoura, Egypt.*

**Corresponding author.**

Salma Allam

Faculty of Medicine, Galala University, Suez, Egypt.

+201501000234

[Salma.allam@gu.edu.eg](mailto:Rahma.mgahed00361@med.aun.edu.eg)

**Supplementary Appendix “Daytime and Nighttime Glycemic Control with Control-IQ Technology vs. Standard Therapy in Type 1 Diabetes: A Systematic Review and Meta-Analysis with Trial Sequential Analysis and GRADE Assessment”**

Contents

[**Supplementary figure 1:** Risk of bias assessment. 4](#_Toc204136460)

[**Supplementary figure 2:** Nighttime Time in the range 70-180mg dl sensitivity analysis. 5](#_Toc204136461)

[**Supplementary figure 3:** Time in the range 70-140mg dl sensitivity analysis. 6](#_Toc204136462)

[**Supplementary Figure 4:** Time below range 70mg dl sensitivity analysis. 7](#_Toc204136463)

[**Supplementary figure 5**: (a) Time below range 54mg/dl, (b) Nightime time below range 54mg/dl, (c) Daytime time below range 54mg/dl. 8](#_Toc204136464)

[**Supplementary figure 6:** Time below range 54mg/dl sensitivity analysis. 9](#_Toc204136465)

[**Supplementary figure 7:** (a) Time above range 180mg/dl, (b) Nighttime Time above range 180mg/dl, (c) Daytime Time above range 180mg/dl. 10](#_Toc204136466)

[**Supplementary figure 8**: Nighttime time above range 180mg/dl sensitivity analysis. 11](#_Toc204136467)

[**Supplementary figure 9:** (a) Time above range 300mg/dl, (b) Nighttime time above range 300mg/dl, (c) Daytime time above range 300mg/dl. 12](#_Toc204136468)

[**Supplementary figure 10**: (a) Coefficient variation CV, (b) Nighttime coefficient variation CV, (c) Daytime coefficient variation, (d)Mean glucose, (e) Low blood glucose index (LBGI), (f) High blood glucose index (HBGI). 13](#_Toc204136469)

[**Supplementary figure 11:** Nighttime coefficient variation CV sensitivity. 14](#_Toc204136470)

[**Supplementary figure 12:** Low blood glucose index LBGI sensitivity analysis. 15](#_Toc204136471)

[**Supplementary figure 13:** High blood glucose index HBGI sensitivity analysis. 16](#_Toc204136472)

[**Supplementary figure 14**: (a) HbA1C level %, (b) Number of patients who achieved HbA1C less than 7% at the end of the trial. 17](#_Toc204136473)

[**Supplementary Figure 15:** (a) Diabetic ketoacidosis (DKA), (b) Severe hypoglycemia. 18](#_Toc204136474)

[**Supplementary figure 16:** Sensitivity analysis TIR 70-180mg/dl by excluding Forlenza 2019 and Ekhlaspour 2019. 19](#_Toc204136475)

[**Supplementary figure 17:** TIR 70-180 mg/dl Sensitivity analysis by excluding Renard 2023. 20](#_Toc204136476)

[**Supplementary figure 18:** trial sequential analysis of TBR 70 mg/dl, using random effect model (DL). The required infromation size to detect or reject the mean differenece of -0.42 (C.I: -0.81 to -0.03) reduction has been 1102 patients using the diversity found in the meta anlysis of 72%, with a double sided α of 0.05 and a β of 0.20 (power of 80.0%) the cumulative z-line (blue line) didn’t cross niether convetional statistical siginificance or (RIS) such indicates that the observed diffenrece between Control IQ and control could not be conclusive and more trials are needed. 21](#_Toc204136477)

[**Supplementary figure 19**: Mean glucose trail sequential analysis, using random effect model(DL), the (RIS) to detect or reject the mean differenece of -15.98 (C.I: -26.24 to -5.72) reduction has been 413 patients using the diversity found in the meta anlysis of 68%, with a double sided α of 0.05 and a β of 0.20 (power of 80.0%) The cumulative Z- curve (blue full line) passed Superiority boundary sample line which suggest that the cumulative evidence is sufficient to conclude that Control IQ is superior to control group with the existing evidence. 22](#_Toc204136478)

[**Supplementary table 1:** Search strategies for all databases: 23](#_Toc204136479)

[**Supplementary table 2**: time endpoints of 24-hour, daytime and nighttime data across the included trials. 24](#_Toc204136480)

[**Supplementary table 3:** Control-IQ compared to standard therapy in type 1 diabetes GRADE assessment of 24 hour data: 25](#_Toc204136481)

[**Supplementary table 4:** Control-IQ compared to standard therapy in type 1 diabetes GRADE assessment of nighttime data: 28](#_Toc204136482)


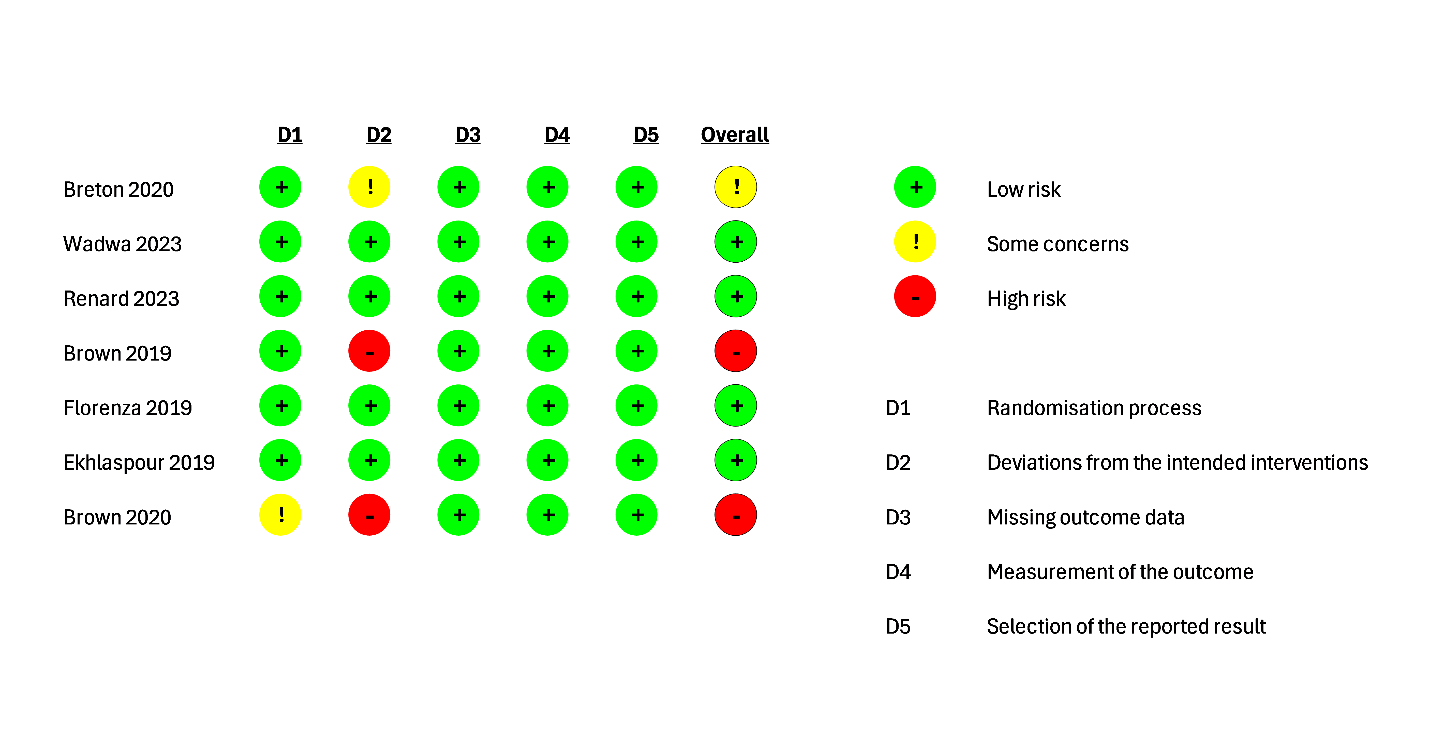


# **Supplementary figure 1:** Risk of bias assessment.


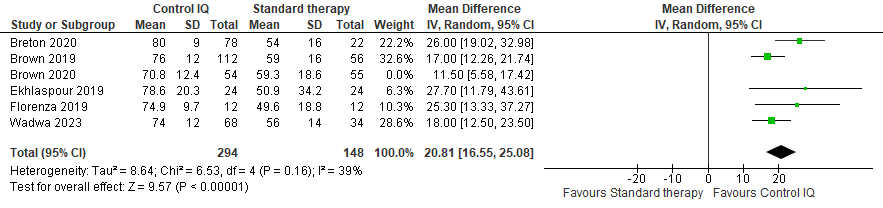


# **Supplementary figure 2:** Nighttime Time in the range 70-180mg dl sensitivity analysis.





# **Supplementary figure 3:** Time in the range 70-140mg dl sensitivity analysis.


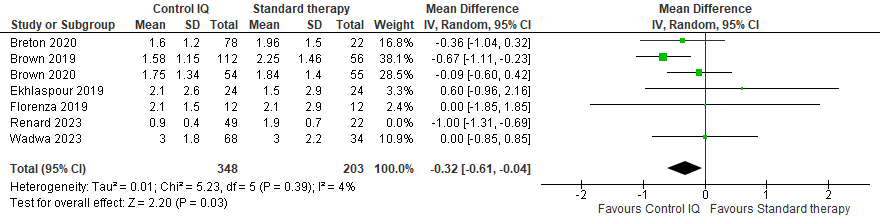


# **Supplementary Figure 4:** Time below range 70mg dl sensitivity analysis.


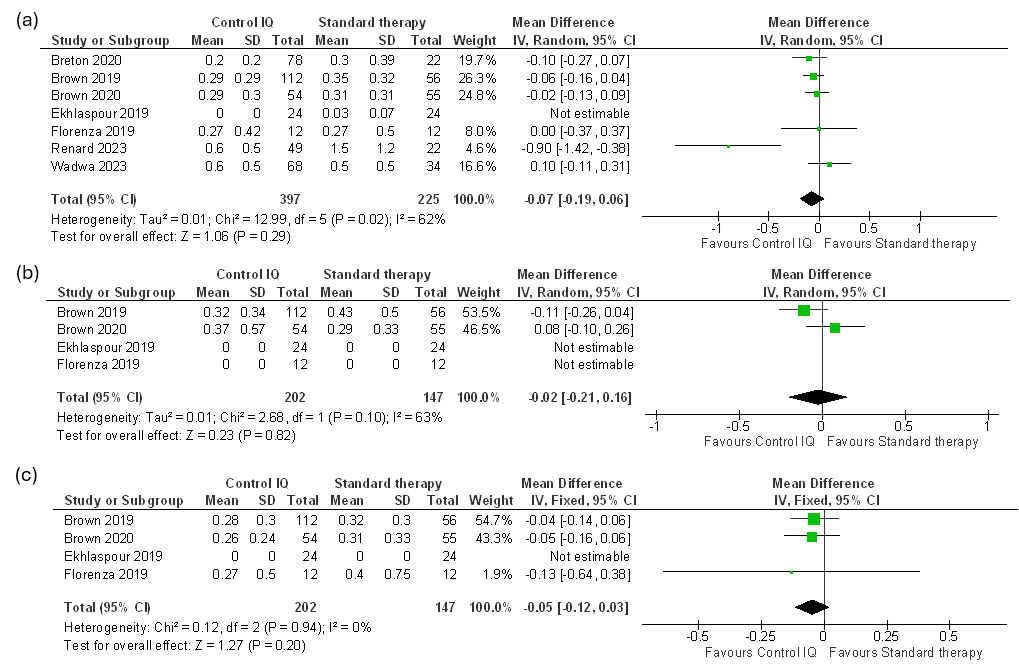


# **Supplementary figure 5**: (a) Time below range 54mg/dl, (b) Nightime time below range 54mg/dl, (c) Daytime time below range 54mg/dl.


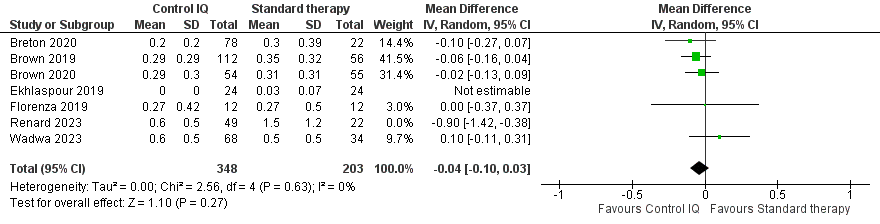


# **Supplementary figure 6:** Time below range 54mg/dl sensitivity analysis.


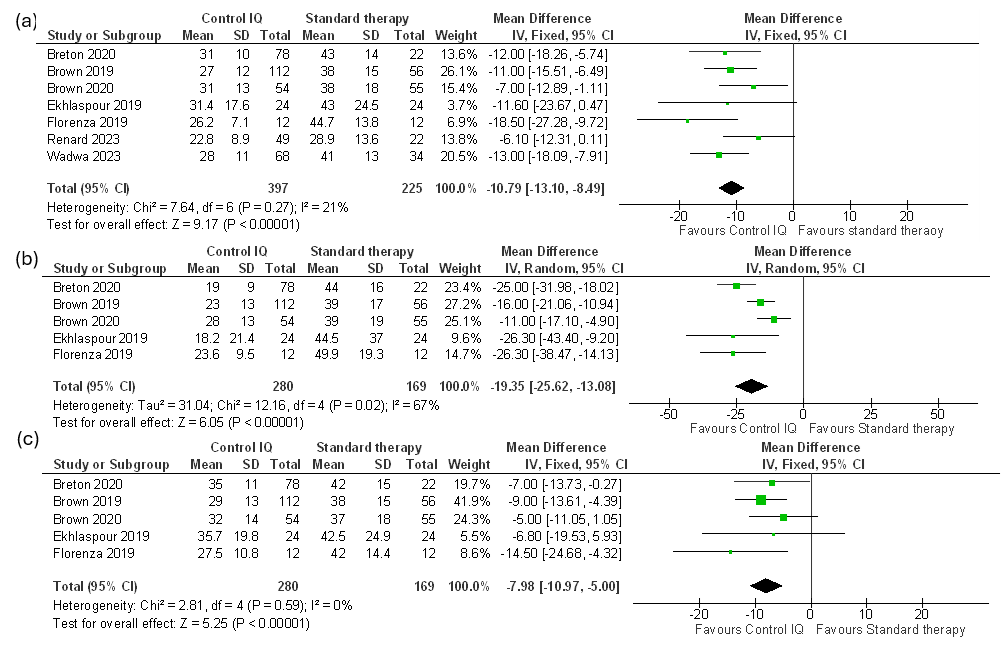


# **Supplementary figure 7:** (a) Time above range 180mg/dl, (b) Nighttime Time above range 180mg/dl, (c) Daytime Time above range 180mg/dl.


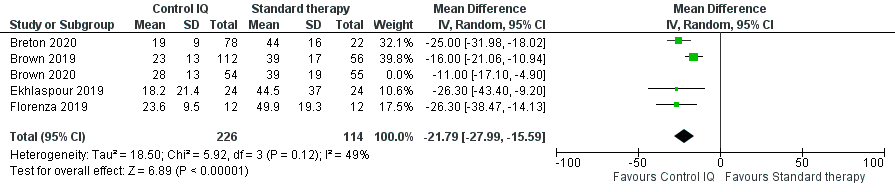


# **Supplementary figure 8**: Nighttime time above range 180mg/dl sensitivity analysis.


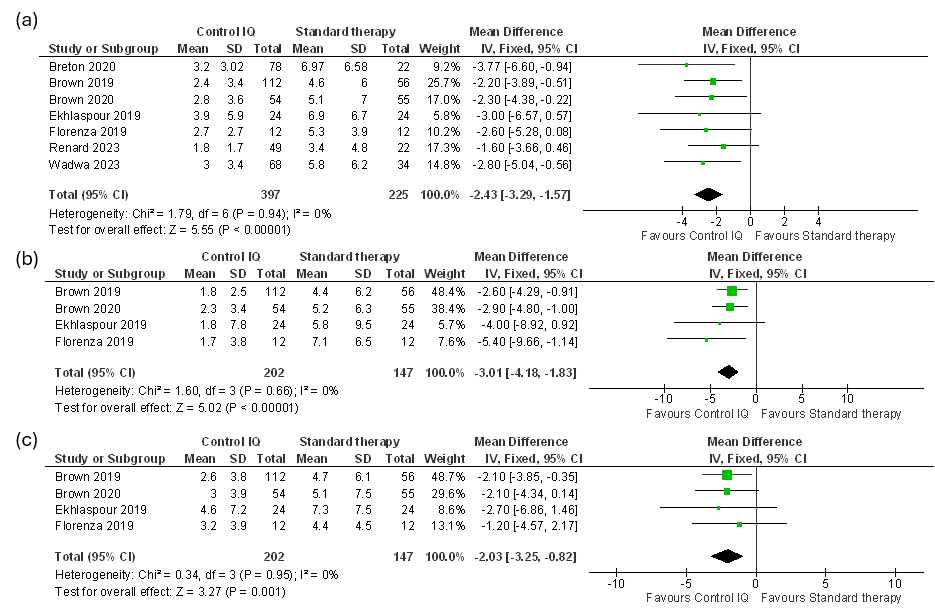


# **Supplementary figure 9:** (a) Time above range 300mg/dl, (b) Nighttime time above range 300mg/dl, (c) Daytime time above range 300mg/dl.


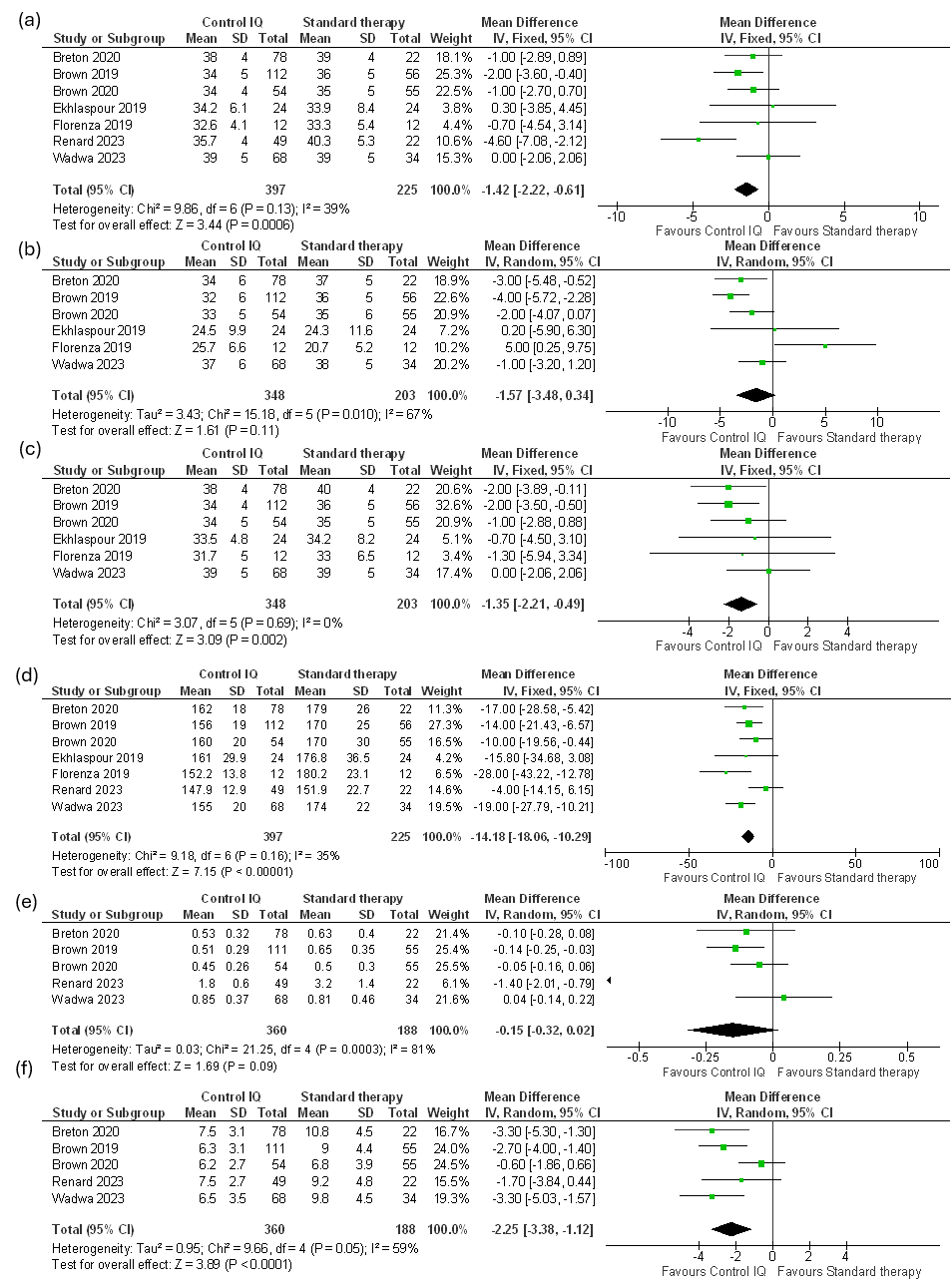


# **Supplementary figure 10**: (a) Coefficient variation CV, (b) Nighttime coefficient variation CV, (c) Daytime coefficient variation, (d)Mean glucose, (e) Low blood glucose index (LBGI), (f) High blood glucose index (HBGI).





# **Supplementary figure 11:** Nighttime coefficient variation CV sensitivity.





# **Supplementary figure 12:** Low blood glucose index LBGI sensitivity analysis.





# **Supplementary figure 13:** High blood glucose index HBGI sensitivity analysis.


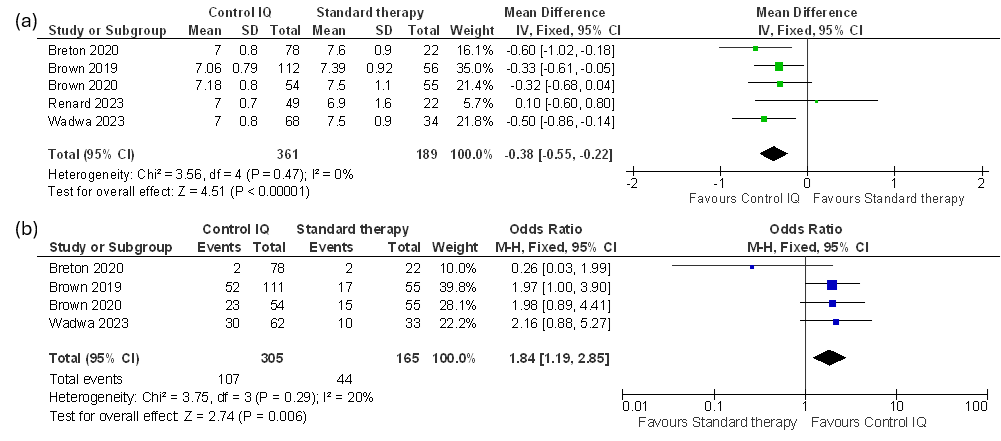


# **Supplementary figure 14**: (a) HbA1C level %, (b) Number of patients who achieved HbA1C less than 7% at the end of the trial.


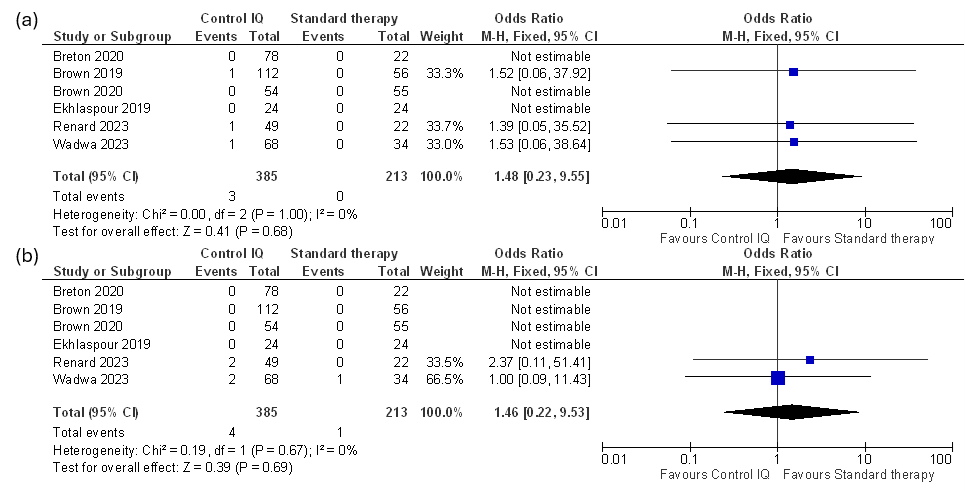


# **Supplementary Figure 15:** (a) Diabetic ketoacidosis (DKA), (b) Severe hypoglycemia.


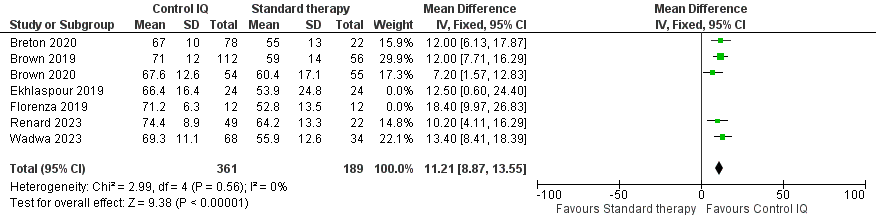


# **Supplementary figure 16:** Sensitivity analysis TIR 70-180mg/dl by excluding Forlenza 2019 and Ekhlaspour 2019.


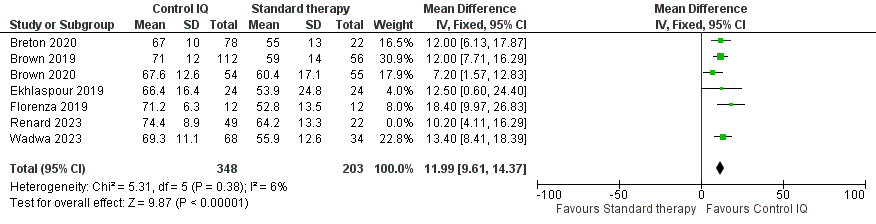


# **Supplementary figure 17:** TIR 70-180 mg/dl Sensitivity analysis by excluding Renard 2023.


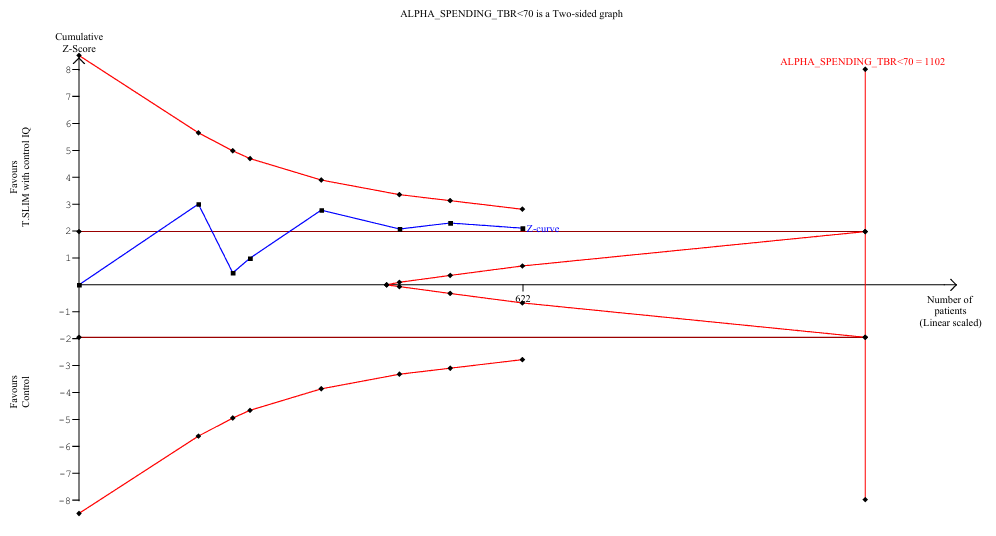


# **Supplementary figure 18:** trial sequential analysis of TBR 70 mg/dl, using random effect model (DL). The required infromation size to detect or reject the mean differenece of -0.42 (C.I: -0.81 to -0.03) reduction has been 1102 patients using the diversity found in the meta anlysis of 72%, with a double sided α of 0.05 and a β of 0.20 (power of 80.0%) the cumulative z-line (blue line) didn’t cross niether convetional statistical siginificance or (RIS) such indicates that the observed diffenrece between Control IQ and control could not be conclusive and more trials are needed.


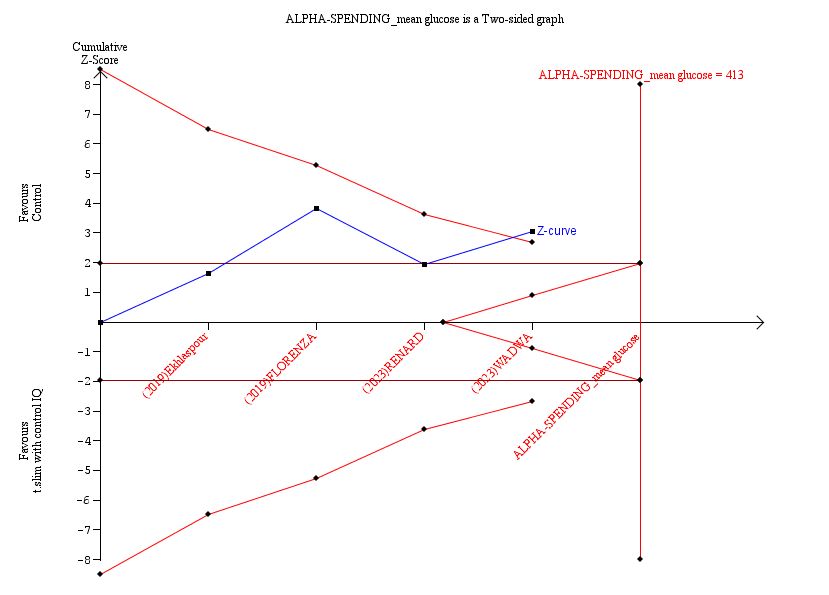


# **Supplementary figure 19**: Mean glucose trail sequential analysis, using random effect model(DL), the (RIS) to detect or reject the mean differenece of -15.98 (C.I: -26.24 to -5.72) reduction has been 413 patients using the diversity found in the meta anlysis of 68%, with a double sided α of 0.05 and a β of 0.20 (power of 80.0%) The cumulative Z- curve (blue full line) passed Superiority boundary sample line which suggest that the cumulative evidence is sufficient to conclude that Control IQ is superior to control group with the existing evidence.

# **Supplementary table 1:** Search strategies for all databases:

| **Database** | **Search strategy** | **Number of citations** |
| --- | --- | --- |
| **PubMed** | Tandem Control-IQ"[All Fields] OR ("t slim"[All Fields] AND "X2"[All Fields] AND ("insulin"[MeSH Terms] OR "insulin"[All Fields] OR "insulin s"[All Fields] OR "insuline"[All Fields] OR "insulinic"[All Fields] OR "insulinization"[All Fields] OR "insulinized"[All Fields] OR "insulins"[MeSH Terms] OR "insulins"[All Fields]) AND "Pump"[All Fields]) OR (("advance"[All Fields] OR "advanced"[All Fields] OR "advancement"[All Fields] OR "advancements"[All Fields] OR "advances"[All Fields] OR "advancing"[All Fields]) AND "hybrid-closed"[All Fields] AND "loop"[All Fields]) OR "Control-IQ"[All Fields] OR "AHCL"[All Fields] | 318 |
| **Scopus** | TITLE-ABS-KEY ( "Tandem Control-IQ" OR "t:slim X2 Insulin Pump" OR "advanced hybrid-closed loop" OR "Control-IQ" OR "AHCL" ) | 362 |
| **Web of Science** | "Tandem Control-IQ" OR "t:slim X2 Insulin Pump" OR (advanced hybrid-closed loop) OR "Control-IQ" OR "AHCL" (Topic) | 460 |
| **Cochrane library** | "Tandem Control-IQ" OR "t:slim X2 Insulin Pump" OR (advanced hybrid-closed loop) OR "Control-IQ" OR "AHCL" | 199 |

| **Study ID** | **24 hour period** | **Nighttime** | **Daytime** |
| --- | --- | --- | --- |
| **Forlenza 2019** | 24 hour | 11 PM to 7 AM | 7 AM to 11 PM |
| **Brown 2019** | 24 hour | 12 AM to 6 AM | 6 AM to 12 PM |
| **Ekhlaspour 2019** | 24 hour | 11 PM to 7 AM | 7 AM to 11 PM |
| **Renard 2023** | 24 hour | _- | _- |
| **Wadwa 2023** | 24 hour | 10 PM to 6 AM | 6 AM to 10 PM |
| **Brown 2020** | 24 hour | 12 AM to 6 AM | 6 AM to 12 PM |
| **Breton 2020** | 24 hour | 12 AM to 6 AM | 6 AM to 12 PM |
|  | **Severe hypoglycemia definitions** | | |
| **Forlenza 2019** | - | | |
| **Brown 2019** | Severe hypoglycemia: was defined as hypoglycemia leading to the need for assistance because of altered consciousness. | | |
| **Ekhlaspour 2019** | Severe hypoglycemia had no clear definition | | |
| **Renard 2023** | Severe hypoglycemia: a hypoglycemic event requiring assistance of another person to actively administer carbohydrate, glucagon, or take other corrective actions due to altered consciousness, seizures, or loss of consciousness. | | |
| **Wadwa 2023** | Severe hypoglycemia: an event requiring assistance from another person to actively administer carbohydrate, glucagon, or take other corrective actions | | |
| **Brown 2020** | Severe hypoglycemia: as an event associated with altered mental status and requiring assistance for recovery | | |
| **Breton 2020** | Severe hypoglycemia: a hypoglycemia leading to the need for assistance because of altered consciousness | | |

# **Supplementary table 2**: time endpoints of 24-hour, daytime and nighttime data across the included trials.

# **Supplementary table 3:** Control-IQ compared to standard therapy in type 1 diabetes GRADE assessment of 24 hour data:

|  | | | | | | | | | | | |
| --- | --- | --- | --- | --- | --- | --- | --- | --- | --- | --- | --- |
| **Certainty assessment** | | | | | | | **Summary of findings** | | | | |
| **Participants (studies) Follow-up** | **Risk of bias** | **Inconsistency** | **Indirectness** | **Imprecision** | **Publication bias** | **Overall certainty of evidence** | **Study event rates (%)** | | **Relative effect (95% CI)** | **Anticipated absolute effects** | |
|  |  |  |  |  |  |  | **With Standard therapy** | **With Control-IQ** |  | **Risk with Standard therapy** | **Risk difference with Control-IQ** |
| **TIR 70-180 mg/dl** | | | | | | | | | | | |
| 622 (7 RCTs) | not serious | not serious | not serious | not serious | publication bias strongly suspected^a^ | ⨁⨁⨁◯ Moderate^a^ | 225 | 397 | - | - | MD **11.75 higher** (9.54 higher to 13.97 higher) |
| **TIR 70-140 mg/dl** | | | | | | | | | | | |
| 547 (6 RCTs) | not serious | not serious | not serious | not serious | publication bias strongly suspected^a^ | ⨁⨁⨁◯ Moderate^a^ | 201 | 346 | - | - | MD **10.02 higher** (6.71 higher to 13.32 higher) |
| **TBR <70 mg/dl** | | | | | | | | | | | |
| 622 (7 RCTs) | not serious | not serious | not serious | not serious | publication bias strongly suspected^a^ | ⨁⨁⨁◯ Moderate^a^ | 225 | 397 | - | - | MD **0.42 lower** (0.81 lower to 0.03 lower) |
| **TBR < 54 mg/dl** | | | | | | | | | | | |
| 622 (7 RCTs) | serious^b^ | not serious | not serious | serious^c^ | publication bias strongly suspected^a^ | ⨁◯◯◯ Very low^a,b,c^ | 225 | 397 | - | - | MD **0.07 lower** (0.19 lower to 0.06 higher) |
| **TAR 180 mg/dl** | | | | | | | | | | | |
| 622 (7 RCTs) | not serious | not serious | not serious | not serious | publication bias strongly suspected^c^ | ⨁⨁⨁◯ Moderate^c^ | 225 | 397 | - | - | MD **10.79 lower** (13.1 lower to 8.49 lower) |
| **TAR > 300 mg/dl** | | | | | | | | | | | |
| 622 (7 RCTs) | not serious | not serious | not serious | not serious | publication bias strongly suspected^a^ | ⨁⨁⨁◯ Moderate^a^ | 225 | 397 | - | - | MD **2.43 lower** (3.29 lower to 1.57 lower) |
| **Glucose CV** | | | | | | | | | | | |
| 622 (7 RCTs) | serious^b^ | not serious | not serious | not serious | publication bias strongly suspected^a^ | ⨁⨁◯◯ Low^a,b^ | 225 | 397 | - | - | MD **1.42 lower** (2.22 lower to 0.61 lower) |
| **LBGI** | | | | | | | | | | | |
| 548 (5 RCTs) | serious^b^ | not serious | not serious | serious^c^ | publication bias strongly suspected^a^ | ⨁◯◯◯ Very low^a,b,c^ | 188 | 360 | - | - | MD **0.15 lower** (0.32 lower to 0.02 higher) |

**CI:** confidence interval; **MD:** mean difference

**Explanations**

a. asymmetrical distribution of the studies on the funnel plot.

b. Two studies, contributing to 50% or more of the analysis weight, showed high risk of bias.

c. A wide confidence interval that does not exclude the appreciable harm or benefit.

# **Supplementary table 4:** Control-IQ compared to standard therapy in type 1 diabetes GRADE assessment of nighttime data:

|  | | | | | | | | | | | |
| --- | --- | --- | --- | --- | --- | --- | --- | --- | --- | --- | --- |
| **Certainty assessment** | | | | | | | **Summary of findings** | | | | |
| **Participants (studies) Follow-up** | **Risk of bias** | **Inconsistency** | **Indirectness** | **Imprecision** | **Publication bias** | **Overall certainty of evidence** | **Study event rates (%)** | | **Relative effect (95% CI)** | **Anticipated absolute effects** | |
|  |  |  |  |  |  |  | **With Standard therapy** | **With Control IQ** |  | **Risk with Standard therapy** | **Risk difference with Control IQ** |
| **TIR 70-180 mg/dl** | | | | | | | | | | | |
| 551 (6 RCTs) | not serious | not serious | not serious | not serious | publication bias strongly suspected^a^ | ⨁⨁⨁◯ Moderate^a^ | 203 | 348 | - | - | MD **19.29 higher** (14.6 higher to 23.98 higher) |
| **TIR 70-140 mg/dl** | | | | | | | | | | | |
| 301 (3 RCTs) | serious^b^ | not serious | not serious | not serious | publication bias strongly suspected^a^ | ⨁⨁◯◯ Low^a,b^ | 123 | 178 | - | - | MD **14.72 higher** (5.93 higher to 23.5 higher) |
| **TBR 70 mg/dl** | | | | | | | | | | | |
| 551 (6 RCTs) | serious^b^ | not serious | not serious | not serious | publication bias strongly suspected^a^ | ⨁⨁◯◯ Low^a,b^ | 203 | 348 | - | - | MD **0.57 lower** (0.9 lower to 0.23 lower) |
| **TBR 54 mg/dl** | | | | | | | | | | | |
| 349 (4 RCTs) | serious^b^ | not serious | not serious | serious^c^ | none | ⨁⨁◯◯ Low^b,c^ | 147 | 202 | - | - | MD **0.02 lower** (0.21 lower to 0.16 higher) |
| **TAR 180 mg/dl** | | | | | | | | | | | |
| 449 (5 RCTs) | serious^b^ | not serious | not serious | not serious | publication bias strongly suspected^a^ | ⨁⨁◯◯ Low^a,b^ | 169 | 280 | - | - | MD **19.35 lower** (25.62 lower to 13.08 lower) |
| **TAR 300** | | | | | | | | | | | |
| 349 (4 RCTs) | serious^b^ | not serious | not serious | not serious | publication bias strongly suspected^a^ | ⨁⨁◯◯ Low^a,b^ | 147 | 202 | - | - | MD **3.01 lower** (4.18 lower to 1.83 lower) |
| **Glucose CV** | | | | | | | | | | | |
| 551 (6 RCTs) | not serious | not serious | not serious | serious^c^ | publication bias strongly suspected^b^ | ⨁⨁◯◯ Low^b,c^ | 203 | 348 | - | - | MD **1.57 lower** (3.48 lower to 0.34 higher) |

**CI:** confidence interval; **MD:** mean difference

**Explanations**

a. asymmetrical distribution of the studies on the funnel plot.

b. Two studies, contributing to 50% or more of the analysis weight, showed high risk of bias.

c. A wide confidence interval that does not exclude the appreciable harm or benefit.
